# Mass Gathering Medicine in Soccer Leagues: A Review and Creation of the SALEM Tool

**DOI:** 10.3390/ijerph18199973

**Published:** 2021-09-22

**Authors:** Anas A. Khan, Abdulrahman Y. Sabbagh, Jamie Ranse, Michael S. Molloy, Gregory R. Ciottone

**Affiliations:** 1 Department of Emergency Medicine, College of Medicine, King Saud University, Riyadh 12372, Saudi Arabia; 2 The Global Centre for Mass Gatherings Medicine, Ministry of Health, Riyadh 12372, Saudi Arabia; 3 Emergency Medicine Administration, King Fahad Medical City, Riyadh 12231, Saudi Arabia; aysabbagh.em@gmail.com; 4 Menzies Health Institute Queensland, Griffith University, Gold Coast 4215, Australia; j.ranse@griffith.edu.au; 5 Department of Emergency Medicine, Gold Coast Health, Gold Coast 4215, Australia; 6 University College Dublin School of Medicine and Medical Science, D04 V1W8 Dublin, Ireland; mickmolloy@mac.com; 7 Faculty Sports and Exercise Medicine, Royal College of Surgeons in Ireland, RCSI House 121 St. Stephen’s Green, D02 H903 Dublin, Ireland; 8 Disaster Medicine Fellowship, Beth Israel Deaconess Medical Center, 457 Brookline Ave., Boston, MA 02215, USA; 9 Wexford General Hospital, Ireland East Hospital Group, Carricklawn, Y35 Y17D Wexford, Ireland; 10 Harvard Medical School, Boston, MA 02115, USA; gciotton@bidmc.harvard.edu

**Keywords:** football events, mass gathering, public health risks, overcrowding, risk assessment

## Abstract

Potential risks for public health incidents, outbreaks, and casualties are inferred at association football events, especially if event organizers have not taken appropriate preventative measures. This review explores the potential risks imposed by mass gathering (MG) football events, with particular emphasis on tools and methodologies to manage the risks of football MG events. Effective planning and implementation of MGs along with the mitigation of risks related to people’s health require special attention to all potential threats, especially in frequent and recurring MG events such as football leagues. The well-being of all participants can be compromised by ignoring a single risk. Healthcare systems should cooperate with all stakeholders and organizations who are involved in MG management and response. Provision of services during MG or a disaster must be performed by trained personnel or entities that have full access to available resources in accessible publicly known locations at the MG event site. Several MG assessment tools were developed worldwide; however, to adapt to the Saudi context, SALEM tool was developed to provide a guide for MG planning and assessment. SALEM assesses the risks of MG events with scores that help to categorize the risk of MG events by offering recommendations for required resources.

## 1. Introduction

Public mass gatherings (MGs) carry various health risks and represent a challenging concern for entities providing emergency medical services [1]. Due to overcrowding at MG events, there are potential risks for public health incidents, outbreaks, and casualties, especially if event organizers have not taken proper measures [2]. Healthcare systems are designed to provide routine medical services, often with limited capacity to expand. Therefore, MG events can put a strain on healthcare service providers. The World Health Organization (WHO) defines a mass gathering event as “an organized or unplanned event where the number of people attending is sufficient to strain the planning and response resources of the community, state or nation hosting the event” [1]. The definition of the WHO is intentionally not related to the scale of the MG event or the number of participants, although these factors certainly affect the risk management process. However, each venue has a different capacity to handle large crowds, with some facilities, such as airports or markets, handling up to 100,000 people daily with minimum complications [2,3,4,5,6]. The goals of healthcare systems in MG events are to provide services to the attendees, prevent and reduce injuries, prevent and reduce disease infection risks, increase the level of safety among participants, and ensure the sustainability of timely routine and emergent medical care to the general population during the MG event. To reach these objectives, healthcare systems are faced with immense and complicated challenges [7]. For proper management of MG events, potential health risks should be recognized as the first step through the performance of a comprehensive risk assessment [8]. This narrative review explores the potential risks imposed by MG football events, with particular emphasis on tools and methodologies to manage risks. The objective of this article is to highlight and evaluate the risks of football MG events, provide a reference standard for regulating events, and minimize health risks through recommended steps to enhance the safety and well-being of participants.

## 2. Healthcare Risks of Football MG Events

Sporting MG events are typically characterized by energetic and sometimes aggressive spectators, which can increase the risks of injuries, violence, and cardiovascular events [1]. Jones et al. proposed that the Olympic Games inspired the foundation of sports medicine, and later the science of MG medicine, created by the experience of managing public health at these large events [9]. It is considered a privilege for countries to host the Olympic Games. Nations work intensively to ensure an incident-free event by assessing the facilities for the MG events, the planned response of their local healthcare systems, and their disaster response plans [10]. Sporting events can attract a very large number of participants and audience. For instance, the Boston Marathon attracts 500,000 fans each year, and includes more than 36,000 participants [11].

Unlike marathons, however, football events result in mass spectatorship of large audiences and fans. Fans are mainly in the stadium seating and, in some tournaments, in the fan zones outside the stadium. Due to its global reach and appeal, football is played by teams from 300,000 clubs worldwide, with more than five million referees, assistant referees, and officials directly involved [10,12]. Football events may resemble a hybrid of sporting and religious MG events, especially with devoted fans and followers [10]. Football games and competitions involve large numbers of supporters located in the confined environment of a stadium, with audiences having varying levels of health status and needs that should be considered when planning a football match or tournament [13].

The main potential public health concerns associated with MG events, including football events, range from infectious diseases to injuries, traffic accidents, heat-related illnesses, insect stings, non-communicable diseases, and terrorism [14]. In a recent systematic review by Tavan et al., it was reported that the potential health risks in MG events can be classified into five domains based on risk type: public health, environmental, individual, psychological, and management risk domains [8].

One of the most important risks associated with football MG events, especially after the COVID-19 pandemic, is the communicable disease risk. Outbreaks and the spread of infectious or communicable diseases at football MG events represent a major concern for global health security, especially if combined with the absence of infection prevention and control strategies [15,16,17]. Control of communicable diseases in football MG events is of utmost importance to avoid the super-spread of any infection outbreak from the MG event to the entire population. The role of MG events in the dissemination of pathogens and the spread of antimicrobial resistance globally, especially in international MG events such as the World Cup tournament or the Olympics, has been of particular interest, especially with emerging infections [18,19,20]. Notably, it was reported that some football events were considered to be “biological bombs” that helped in the wide spread of COVID-19 [21,22,23,24].

Football MG events are also more prone to mass fatality incidents [25], Table 1. Environmental factors, such as extreme weather conditions, threaten the well-being of participants at football MG events. The population density in certain events is also a main threat, which may cause death and various injuries due to population pressure, trampling and stampede, and other actions that will not only cause higher morbidity and mortality but also may hinder the access of emergency response teams for adequate access and egress at the MG events [8,26,27,28,29]. Moreover, heat exhaustion, dehydration, and sunburns are also a common risk in football MG events, especially as these events are mainly performed outdoors [30]. On an individual level, the health risk imposed by MG events depends on the participants’ age, health condition (whether physiological such as pregnancy or pathological such as chronic diseases), and other factors that render the individual more vulnerable to morbidity or mortality [27,31]. As for the psychological state of the participants, the aggressive and energetic behavior of the crowd imposes a higher risk for injuries or other incidents in MG events [8,32,33,34].

Regarding the management of football MG risks, lack of resources, lack of competent staff, the ineffectiveness of MG safety measures, issues of communication or coordination, difficult access to medical facilities, and prolonged duration of the MG event are all identified as potential factors in MGs that may increase the health risks and the workload of emergency services [15,53,54,55,56]. The availability of qualified medical staff in football MGs, including doctors, nurses, and emergency technicians, is one of the factors mitigating these health risks, by providing more timely medical service and preventing unnecessary dispatch of ambulances [25].

Ineffective risk communication is another contributing factor for health-related events during football MGs. Risk communication is a core component of preparedness and response to MG events, defined as “the exchange of real-time information, advice, and opinions between experts and people facing threats to their health, economic, or social well-being” [57]. According to WHO guidance, systematic, ongoing MG risk communication should be an essential part of regular MG event planning, rather than being a crisis communication plan. As well, risk communication should not be exclusively based on information transmission, but should encompass an organized plan to guide governance decisions, policies, and practices, in the context of cultural, social, political, and economic dimensions and settings [58]. Effective risk communication represents the cornerstone for a risk management strategy aiming to improve how people perceive health risks during pandemics, and how they react to healthcare emergencies and adhere to self-protective measures [59].

## 3. Influential Factors of the Healthcare System’s Response

MG risks are directly related to the current threats, degree of exposure, and vulnerability of the population and environment [2,60]. Demographics of MG event attendees might influence the degree of potential risk at an MG event, such as age (elderly participants are more vulnerable to health-related risks), participants with a history of comorbidities, and co-existing physiological health conditions such as pregnancy. Influential factors must be taken into consideration while planning for an event, including location, altitude, weather conditions, duration of the event, crowd behavior, the nature of the event (whether it is considered a local or national or international event), staff qualification, level of preparedness, gathering size, population density, amount of health facilities, and concurrent epidemics or pandemics. Equally important is the nature of the event—local, national, or international attendees. Moreover, the risk of communicable infectious diseases rises with increasing population density. Increasing expansion of MG events and the number of people involved would also increase risks, with mobile populations having been shown to produce more emergency service workloads than seated populations [27,61,62,63].

## 4. MG in Football and History of Disasters

Football events are one of the most commonly held MG events. Previous literature reported numerous incidents that added to the body of knowledge of MG lessons learned (Table 1). Although previous MG disasters have been resulted in much morbidity and mortality, they have yet provided areas for improvement to the community and healthcare systems. Throughout history, governments and healthcare systems have learned how to improve football event assessments, including controlling and preventing overcrowding, providing access to emergency medical services, ensuring fire safety measures and plans, securing onsite medical preparedness, and ready transportation for emergency and critical cases [25]. In 1971, a football match in Glasgow, United Kingdom (UK) caused more than 200 casualties, resulting from a crush between fans entering and exiting. This incident highlighted the urgent need for crowd control and the directional flow of fans [64]. A 1986 fire after a football match in Bradford, UK that resulted in more than 290 casualties highlighted the need for onsite medical services, in addition to robust and effective safety precautions [65]. One of the most important after-action analyses was the “Taylor Report” following the overcrowding disaster that resulted in more than 850 casualties in a football match in Sheffield, UK. The report recommended all-seated stadia remove of potentially dangerous barriers and improve stadia medical facilities [66]. In Ellis Park, South Africa, the police used tear gas to control the crowd, not considering that this action would cause more than 40 deaths due to a human stampede [47].

## 5. Impact of the COVID-19 Pandemic on Football

The COVID-19 pandemic has disrupted football events worldwide, with the UEFA EURO 2020 tournament being postponed to 2021. Across the world and to varying degrees, leagues and competitions have been canceled or postponed [67]. Despite the WHO declaration of the “Pandemic state”, surprisingly, many football leagues worldwide continued the regular league schedules with regular audience attendance. On 19 February 2020, a European Champions League match was held in Italy and was attended by 40,000 residents of Bergamo city; the number of COVID-19 cases increased dramatically weeks later, and Bergamo recorded the highest number of confirmed cases amongst different cities of the world. The Italian authority considered this event as a “biological bomb” that helped in widespread dispersion of the virus and crippling of the health service in Northern Italy for a considerable period. However, the contribution of this match to the remarkable rise of COVID-19 cases in the city is controversial, as other reports noted a significant rise in the confirmed cases before the event [21,22,23,24]. The first Italian SERIE A League player diagnosed with COVID-19 occurred on 11 March 2020; however, the first professional SERIE C player was diagnosed in Tuscany on 23 February 2020 [68]. The Italian Series was postponed in March 2020 after several players tested positive for COVID-19, the first time that SERIE A had been postponed since WWII [69]. The rest of the football leagues in Europe were also suspended as a reaction to the pandemic, to ensure the safety of both the players and audience [70,71,72,73]. Both the Confederation of African Football (CAF) Champions League and CAF Cup semi-finals were postponed from their original schedules. Asian football leagues, including the Chinese, Korean, and Japanese leagues, also postponed their competitions. The Saudi Professional Football League was also suspended on 14 March 2021 [74]. According to the Saudi Arabian Football Federation, 1351 PCR tests were carried out for Saudi players and team staff between 21 June and 8 July 2020. A total of 50 players and 47 team staff around the Kingdom tested positive for COVID-19 out of 1351 tests (positive rate = 7.2%) [75].

The WHO has compiled a set of guidance documents and risk assessment tools to facilitate the preparation, organization, and delivery of MGs in response to the COVID-19 pandemic recognizing the fact that MGs pose a significant risk of transmission [76]. The recommendations address the risk of transmission among players, nominating contact sports as high-risk sports; close physical contact among players increases the risk of transmission of COVID-19. WHO recommendations are also directed towards fans; properly planned football events with seated stadia, outdoor nature, and known points of entry and exits enable prevention and control of infection as well as management of other potential MG risks as long as community transmission levels are within tolerable levels [77]. The Saudi Center for Disease Prevention and Control (CDC) published a sport/exercise protocol allowing sports events and training to continue while minimizing infection risk. Briefly, the protocols emphasized the importance of hand sanitizer availability, proper handwashing, immediate disinfection of sports outfits, towels, pads, and surfaces, among other hygienic measures. Clear instructions were also given regarding social distancing measures in sports facilities and crowd avoidance during training and MG events. The instructions also prevent shared clothes and equipment and state that coaches, administrative, and medical staff must wear face masks. Finally, the Saudi protocols emphasize the importance of inspection points at sports facility entrances and advise that suspected cases should be immediately reported and monitored. The Saudi Ministry of Health (MoH) appoints supervisors to ensure protocol implementation for sports activities and requirements are met in the sports facilities [78].

By the end of June 2020, most national federations within the Asian Football Confederation (AFC) announced plans to resume national league football activities, including the Saudi Football Federation (SFF). The SFF announced that the Prince Mohammed bin Salman Cup (Saudi Professional League) would resume starting on 4 August 2020 [79]. The SFF, in collaboration with the Saudi Professional League and governmental sectors, agreed a standard safety protocol in compliance with COVID-19 FIFA guidelines which should be strictly followed before conduction of any training session or competition restart. The protocol includes mandatory appropriately timed pre-match PCR testing of all players, delegates, and officials; appropriate physical distancing between players and staff on the pitch verges; prevention of handshaking or exchange of t-shirts; appropriate cleaning and hygiene regimen; disinfection of all vehicles used to transport teams; and specific protocols for hotels accommodating teams and staff. Similar procedures were applied for all national, regional, and international competitions by the relevant football federations. In September 2020, Saudi Arabia’s Al-Hilal team was disqualified from the Asian Football Confederation Champions League after 31 players and staff had a positive PCR test, which led to the inability to field a full team for the competition [80].

## 6. Preparation and Planning for Football MG Events

Inappropriate management of public and environmental health in football MGs threatens the health and welfare of both participants and staff [8]. A 2017 Saudi study presented at the 3rd International Conference on Mass Gatherings Medicine, Riyadh, Kingdom of Saudi Arabia explored the risk, risk mitigation, and health responses at MGs. The event addressed risk assessment at MGs, risk mitigation, and the concept of resilience in the context of MGs. Health services planning and responses, including during emergencies, were further discussed drawing from experiences in various MGs such as the Hajj, sporting events, and music festivals.

Before any MG event, a comprehensive risk assessment must take place using robust tools. The risk assessment should include an assessment of the event circumstances, taking into account multiple factors such as weather, spectator demographics, and crowd behavior [1]. Suggested factors for the prediction of a low-risk football MG event disaster occurrence include: (1) small crowds (less than one-third of stadium capacity), (2) no violence expected, and (3) no standing spectators (all-seated stadium). The risk of disaster occurrence during football MG events increases with an excess audience number (occupying more than 50% of the stadium capacity), events taking place at night, hot or rainy conditions, or in case of expected violence based on event history or advanced medical intelligence [13]. Risk assessment should also extend to spectator demographics including their behavior and health conditions; children and elderly are more vulnerable, while pregnant women or an audience with high percentage of attendees with chronic diseases are also significant risk factors [32,33].

Health management and major incident planning are also crucial prior to the event. The basic elements of a Stadium Major Incident Plan (MIP) involve preparation of a fully equipped command and control center, safety consideration, triage, treatment, transport, and debriefing [13,81,82]. The MIP should take an all-hazards approach and be adaptable to the type of expected incident(s). Safety planning is the primary element involved with respect to incidents not related to spectators, such as fires, structural collapse, or explosions. When primary spectator-involved incidents are considered, including stampeding crowd masses or violence, medical management and minimization of injury are the primary concern. During a major incident, the number of casualties will dictate the prioritization of patients; this is why triage planning is of prime importance. Treatment and transport planning should consider the capacity, capability, training and availability of both staff and equipment in addition to the preparedness of the healthcare facilities nearest the event. “Hot-wash” and delayed debriefing sessions should be held to study what happened and how to avoid similar future incidents ensuring that involved staff are not adversely affected psychologically [81].

It is necessary to ensure staff training, adequate ticketing system, effective crowd flow management, and public information systems are the basic pillars while planning for an MG event. During MG events, organizers must perform effective and thorough monitoring and reporting, enabling valid emergency/evacuation plans by checking safety systems, emergency exits and fire settings, enabling clear information for attendees, and ensuring exit routes are free from obstacles to facilitate evacuation in case of emergency [1,81]. Having clear plans, effective communication and training, and drills are essential measures for effective emergency response at MGs [20]. The coordination and cooperation plan between major MG event stakeholders, including the MoH and other ministries, must be pre-agreed and published for the event safety coordinators. With respect to international MGs, coordination, cooperation, and communications with other organizations such as the WHO should be considered early in the planning phase [8].

**Figure 1:**
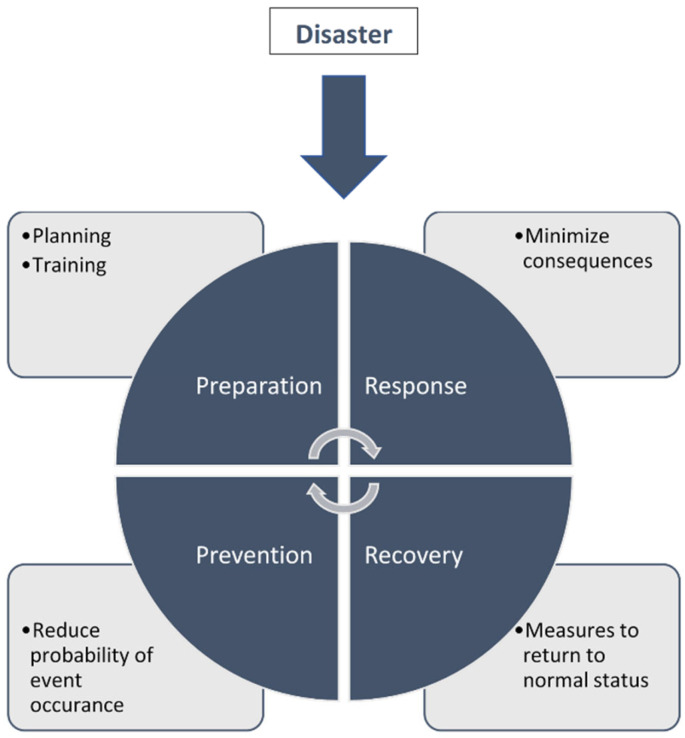
Four disaster management phases (Prevention, Preparation, Response, Recovery).

The disaster management cycle is composed of four phases (Figure 1), and if one is planning a new event, one must start with the prevention phase, aiming to reduce the probability of disaster occurrence by reducing vulnerabilities and enhancing capacities. This phase uses knowledge acquired from previous event history or similar events, lessons learned from MG disasters.

The preparation phase includes appropriate event planning, including pre-event assessment using modeling and simulation for population management and control, early warning systems for environmental risks, as well as possible participants grouping according to demographics or health status [82,83]. In addition, staff selection training and credentialing of staff form a major component of the activities. Risk assessment including checking of infrastructure, facility, safety and security measures, proposed crowd flow control with access/egress modelling (if available), medical preparedness, and emergency response also need to be considered. In this phase, preparedness plans should be tested with all involved parties or leads at least at table-top level but ideally in site walkthrough activities. Plans should also include safe evacuation from venues and access to emergency services, with clearly marked and unobstructed exit routes. Communication plans including prepared clear and concise emergency messaging for participants to prioritize efficient effective evacuation are also necessary. In order to provide efficient health resources and equipment, MG administrators should have reliable mitigation and preparedness data, as most injuries in MGs originate from lack of risk management strategies [7]. Training of staff for mass casualty management including the triage methodologies to be used, treatment priorities and limitations of on-site treatment, transport destinations for the differing triage categories and evacuation routes for emergency medical response teams are vital to the success of the MG event and would benefit from frequent drilling. In case of disaster, the response phase involves proper coordination and implementation of the disaster plan from the preparation phase to minimize the consequences of the disaster. The recovery phase in a MG disaster event cycle is quite different to the normal disaster cycle as the event will most likely have been terminated in most cases and the focus here will be on ensuring that ALL casualties have been accounted for at the venue and that “hot wash” takes place prior to closing up medical activities at the venue. Normal status at MG venues is normally no medical activity in the majority of cases [77,81,84].

For large MG events which may attract international participants a vaccination program based on endemic disease, the participants’ conditions, and other specific factors can reduce the incidence of infectious diseases, especially in international gatherings. In addition to preparation for endemic and seasonal diseases, it is useful to have a preventive plan and disease monitoring system [85].

## 7. SALEM Tool: A Mass Gatherings Risk Assessment Framework

It is widely acknowledged that risk assessment is a critical step during the planning for MG events; such an assessment can help in planning and response activities specific to the event. Risk assessment for a MG includes the evaluation of the potential public health impacts, such as the risk of infectious disease outbreak and injury, thereby determining the systems and processes required to successfully and safely deliver the MG event [1]. Several evidence-based tools were developed over recent years and exhibited fair validity and reliability in properly planning MG events [84].

For example, the Sendai tool was developed as a global tool for risk assessment and disease risk reduction during MG events. The Sendai tool provides a formwork to implement health into disaster risk reduction strategies during MG events planning [2]. However, the tool is mainly concerned with a general preventive-based approach for MG planning, with no specific details on how to classify the health risks during MG or specific details related to sporting MG events. Recently, the MAGRAT (Mass Gathering Risk Assessment Tool) was also recently developed for the preventive-based approach during MG pre-planning phase. The tool was tested in historical real scenarios, including a soccer match, and proved its feasibility. Nonetheless, it is still unclear how the tool can be applied in future sporting MG events [86]. Similar tools were also developed to mitigate the disease risk during MG events in India [87]. In the light of COVID-19 pandemic, The WHO and Cambridge County Council developed two risk assessment tools to assess the health risks during MG events in COVID-19 era. The tools provide risk score and planned actions to mitigate the risk of COVID-19 transmission during MG events. However, none of these tools were specific for sporting events [88,89].

To that extent, the Kingdom of Saudi Arabia has unique experience in utilizing scientific approaches and tools for planning and conducting MG events, which helped in limiting the spread of the previous pandemic such as Middle East Respiratory Syndrome (MERS) [90]. In 2010, the Saudi authority established the Global Center for Mass Gathering Medicine (GCMGM) to ensure proper application of the standards of conducting MG events. Since then, the GCMGM has played a leading role, through hosting of the International Conference on Mass Gathering Medicine, in disseminating the principles of mass gathering medicine, building a collaboration with other mass gathering organizations and WHO-affiliated groups, coordinating policies and procedures, and conducting effective awareness campaigns in this growing field [20,91].

For example, the GCMGM developed the Jeddah tool, based on the Health Emergency and Disaster Risk Management (H-EDRM) framework, for risk assessment of repeated MG events. The tools proved its usefulness in mitigating the risk of diseases during regularly occurring MG events [92].

The GCMGM introduced the “SALEM tool”, which is a mass-gathering risk assessment tool to be implemented in Saudi Arabia. In March 2020, during the peak of COVID-19, the GCMGM introduced a “SALEM COVID-19” tool for assessment of health risks in gatherings and public places, as well as putting forth recommendations for promoting health safety and prevention from COVID-19 infection [93]. In the attempt to create an up-to-date and accurate tool, SALEM was developed based on existing international mass-gathering assessment tools; international guidelines of special events related to health, medical, and safety planning; and Saudi contextual considerations. The SALEM tool evaluates 17 factors that could lead to public health risks (Table 2).

Each of the 17 factors is appointed a score, and MG events are consequently graded based on that score to be either low, medium, or high-risk events/activities (Table 3). According to the risk scores, recommendations are suggested to the event planners for health risk evaluation, management of MG events, and reducing the time between injury and medical interventions, all with the goal of decreasing morbidity and mortality.

Risk assessment tools can pose huge potentials during the planning and conduction of MG events during the pandemic, such as COVID-19. By adopting a structured approach for risk management, public health policymakers can ensure effective implementation and monitoring of key public health principles during MG events. The “SALEM COVID-19” has proven its usefulness during the Hajj 2020. Initially, Saudi Arabia suspended all religious MG events to contain the COVID-19 spread. However, by utilizing the “SALEM COVID-19” tool risk assessment, the Saudi authority successfully conducted a safe Hajj Pilgrimage through an effective adaption of a risk-driven approach [94]. The tool builds a risk assessment model by combining attack rate, country Hajj quota, and global disease severity index [95]. In a recently published report by the GCMGM group, it was estimated that the risk of COVID-19 transmission during Hajj 2020 was huge, both at national and global levels; the report showed that foreign pilgrims would increase the number of infected cases during the gathering, and they would spread the infection to their countries of origin or lead to the development of new unpredictable strains. Moreover, it was estimated that the healthcare services would exceed their maximum capacities when only 10-15% of the average number of pilgrims is reached. The risk assessment model also recommended the exclusion of high-risk groups (e.g., elderly and immunocompromised patients), pre-arrival mandatory PCR testing, two weeks quarantine before and after Hajj rituals, the accommodation of pilgrims in uninhibited remote areas to minimize their interaction with residents, and transportation of the pilgrims in limited cohorts of 30–50 individuals [94].

Thus, the Kingdom issued a decision to down-scale Hajj 2020 for a limited number of pilgrims, including all nationalities residing in Saudi Arabia (divided as 30% Saudi and 70% non-Saudi residents), and executed all of the abovementioned precautionary measures [96]. Fortunately, with the proper precautions such as physical distancing and PPEs, the Kingdom did not record any single case of COVID-19 in Hajj 2020. Of all the pilgrims, healthcare personnel, and nonmedical employees facilitating the rituals, no confirmed cases of COVID-19 were identified during or after Hajj [97]. Such an example reflects how the tool can be useful in curbing the COVID-19 spread.

## 8. Key Insights and Conclusions

Football leagues impose serious risks and public health consequences, especially with the presence of a combination of a high-density audience, restricted points of entry, minimum management of people, and lack of onsite emergency medical care. In the era of the COVID-19 pandemic, football matches could serve as superspreader events that result in serious complications on both national and international levels [25]. In general, health systems are structured to fulfill routine priorities and requirements, and have limited capacity to expand, so MGs often burden the local health care system. Even the most advanced systems and properly planned MG can encounter a disaster that overwhelms local healthcare systems and their capacity to provide an effective emergency response [1]. Effective planning and preparedness for MGs, along with the mitigation of risks related to people’s health require special attention to all potential threats, especially in highly frequent MG events such as football leagues. Effective communication, integrated management, and the well-being of all participants can be compromised by ignoring a single risk [8]. Healthcare systems should share, coordinate, and cooperate with all stakeholders and organizations who are involved in MG management [98]. In addition, understanding crowd psychology and behavior at MGs and during emergencies is essential in determining the most effective strategies for communication with crowds during an MG or during major incidents [20]. Provision of services during MGs or in the event of a disaster must be performed through trained personnel and available resources accessible to staff and spectators in a MG event [81]. Given its unique experience, the FIFA has invested in capacity building, developed strategies for risk communication, utilized robust risk assessment tools, and created both rapid and early response systems that enable early detection of potential risks, and thus a proactive response. Several MG assessment tools were developed worldwide; however, to adapt to the Saudi context, the SALEM tool was developed to provide a guide for MG planning and assessment. SALEM assesses the risks of MG events with scores implicating their public health threats and offers the recommended minimum medical resources needed to ensure the safety of the attendees.

## Figures and Tables

**Table 1:** Mass gathering event disasters in football matches throughout history.

Location and Stadium	Football Event	Incident	Casualties	Date
Bolton, England Burnden Park Stadium [35]	English Football Association Challenge Cup match	A wall collapsed in the stadium before the match crushing fans and sparking a stampede	33 deaths 400 injured	March 1946
Santiago, Chile Estadio Nacional de Chile [36]	The finals match of the South American soccer tournament	Human crush between fans entering the stadium	6 deaths Unknown injuries	March 1955
Lima, Peru The National Stadium [36]	Olympic qualifying match	Human crush and asphyxiation between fans due to overcrowded exiting after police fired tear gas	318 deaths 500 injured	May 1964 *
Kayseri, Turkey Kayseri Atatürk Stadium [37]	Turkish league match	Human crush sparked by stone-throwing and weapon clashes between fans of the two teams	40 deaths 600 injured	September 1967
Buenos Aires, Argentina [38]	First-division league match	Asphyxiation and Human crush against closed Stadium exit between fans unaware of the closed passage	74 deaths 150 injured	June 1968
Glasgow, UK Ibrox Stadium [39]	Football match	Human crush between fans entering and exiting the stadium	66 deaths 140 injured	January 1971
Salvador, Brazil Estádio Fonte Nova [36]	Football match	Human crush sparked by a fight between fans	4 deaths 1500 injured	March 1971
Cairo, Egypt Zamalek stadium [40]	Friendly football match	Human crush due to overcrowding during the influx of fans	49 deaths 50 injured	February 1974
Yaounde, Cameroon [40]	World Cup qualifying match	Mass fight among fans of two teams	2 deaths Unknown injuries	October 1976
Port-au-Prince, Haiti [41]	World Cup qualifying match	Human crush and gunshots sparked by panic after firecracker	6 deaths	December 1976
Piraeus, Greece Karaiskakis Stadium [36]	Derby football match	Human crush among fans exiting stadium through the partially closed exit	21 deaths 55 injured	February 1981
Moscow, Soviet Union Central Lenin Stadium [42]	European Cup match	Human crush and asphyxiation between exiting and returning fans	66 deaths 61 injured	October 1982
Bradford, UK Valley Parade stadium [43]	English league football match	Fire in the Valley Parade stadium	56 deaths 240 injured	May 1985
Brussels, Belgium Heysel Stadium [44]	European Champions Cup Final match	Human crush among Italian fans escaping English fans against a collapsing wall	39 deaths 600 injured	May 1985
Tripoli, Libya Tripoli International Stadium [45]	Football match	Human crush sparked by knife-wielding fan and triggering the collapse of a part of the stadium	20 deaths Unknown injuries	March 1987
Kathmandu, Nepal Dasarath Rangasala Stadium [46]	International football match	Human crush against closed stadium exit sparked by a hailstorm	93 deaths 100 injured	March 1988
Sheffield, UK Hillsborough Stadium [35]	The FA Cup semi-final match	Human crush due to overcrowding during the influx of fans	96 deaths 766 injured	April 1989
Orkney, South Africa Oppenheimer Stadium [47]	A friendly association football match	Human crush among fans escaping from fan brawls	43 deaths 100 injured	January 1991
Bastia, the French island of Corsica Stade Armand Cesari [48]	French Cup semi-final match	Stadium terrace collapse underneath fans before the match	17 deaths 1900 injured	May 1992
Lusaka, Zambia Independence Stadium [45]	World Cup qualifying game.	Human crush during overcrowded fan exit celebrating victory	15 deaths 52 injured	June 1996
Guatemala City, Guatemala Estadio Doroteo Guamuch Flores [45]	World Cup qualifying match	Human crush due to overcrowding during the influx of fans	83 deaths 140 injured	October 1996
Harare, Zimbabwe National Sports Stadium [49]	World Cup qualifying match	Human crush during overcrowded fan exit after police fired tear gas	13 deaths Unknown injuries	July 2000
Salvador, Brazil Estádio Fonte Nova [45]	Local derby match	Upper terrace collapse	7 deaths 10 injuries	2007
Johannesburg, South Africa Ellis Park Stadium [47]	South African league match	Human crush due to overcrowding during the influx of fans	43 deaths Unknown injuries	April 2001
Abidjan, Ivory Coast Stade Félix Houphouët-Boigny [50]	World Cup qualification match	Human crush due to overcrowding of fans before the match after police fired tear gas	20 deaths 135 injuries	March 2009
Port Said, Egypt Port Said Stadium [51]	Egyptian Premier League football match	Human crush among fans exiting the stadium	74 deaths Unknown injuries	February 2012
Kinshasa, Congo Tata Raphaël Stadium [45]	Congo league match	Human crush among fans sparked by police firing tear gas	15 deaths 24 injuries	May 2014
Cairo, Egypt Air Defense Stadium [52]	Egyptian Premier League football match	Human crush due to overcrowding during the influx of fans sparked by police firing tear gas	28 deaths Unknown injuries	February 2015

* Described as the worst disaster in all football history.

**Table 2:** Factors for Risk Scoring in the Saudi SALEM tool.

1	The category of the event (music festivals, exhibitions, or sports competitions, etc.)
2	The expected number of attendees
3	The criteria of attendees (families, sports club fans, community support groups, international stars, or VIP)
4	The nature of attendees’ movements (static audience, young children who need constant monitoring, people with motor disabilities, people who require personal assistance)
5	The age group of attendees
6	The site of the event (open area, specific walled area, inside a building, spacious or narrow area)
7	Available health resources (district hospitals, public hospitals, small hospitals, mobile clinic)
8	The distance to the nearest public or reference hospital
9	Time for the nearest general or reference hospital
10	Duration of the event per day
11	The number of days for the event
12	Possibility of drugs misuse
13	The time of the event
14	The expected temperature at the venue of the event
15	Types of activities in the event (high-risk activities, high competition among participants (ex: wrestling), the interaction between the attendees (for example the final matches), presence of cars or vehicles, including offers or race, presence of fireworks, presence of firearms or flames)
16	Accidents that occurred in previous activities or the same place or expected accidents
17	Food catering services (applying and controlling the specified standards for food catering services, municipality approval is obtained, and valid food catering services are provided)

**Table 3:** Risk classification and preparedness of Saudi SALEM tool.

Low-risk events	Events categorized as low severity recommend risk communication (at the population level), improved monitoring and surveillance, and medical care for the event
Medium-risk events	Medium severity events recommend risk communication (dedicated to the event), active surveillance, medical care for the event, and protective measures for the event (personal protective equipment, handwashing)
High-risk events	High-risk events recommend reducing the number of guests/visitors, adjustment of the crowd flow and seating arrangements, and reducing communication between participants, regulators, and service providers
Severe-risk events	Events with severe risk recommend restructuring the event, changing or moving the event site, postponing or rescheduling the event, or canceling the event
